# Pathogenic variability among *Pasteurella multocida* type A isolates from Brazilian pig farms

**DOI:** 10.1186/s12917-018-1565-2

**Published:** 2018-08-22

**Authors:** João Xavier de Oliveira Filho, Marcos Antônio Zanella Morés, Raquel Rebellato, Jalusa Deon Kich, Maurício Egidio Cantão, Catia Silene Klein, Roberto Maurício Carvalho Guedes, Arlei Coldebella, David Emílio Santos Neves de Barcellos, Nelson Morés

**Affiliations:** 1 Department of Animal Medicine, Universidade Federal do Rio Grande do Sul (UFRGS), Agronomia, Av Bento Gonçalves, 9090, Porto Alegre, Rio Grande do Sul 91540-000 Brazil; 2 Embrapa Suinos e Aves, P.O. Box 121, Concórdia, Santa Catarina 89700-000 Brazil; 3 Preventive Veterinary Medicine Department, Veterinary School, Universidade Federal de Minas Gerais, Belo Horizonte, Brazil

**Keywords:** Respiratory diseases, Pigs, Bronchopneumonia, Polyserositis, *pfhA*, MLST

## Abstract

**Background:**

*Pasteurella multocida* type A (PmA) is considered a secondary agent of pneumonia in pigs. The role of PmA as a primary pathogen was investigated by challenging pigs with eight field strains isolated from pneumonia and serositis in six Brazilian states. Eight groups of eight pigs each were intranasally inoculated with different strains of PmA (1.5 mL/nostril of 10e7 CFU/mL). The control group (*n* = 12) received sterile PBS. The pigs were euthanized by electrocution and necropsied by 5 dpi. Macroscopic lesions were recorded, and swabs and fragments of thoracic and abdominal organs were analyzed by bacteriological and pathological assays. The PmA strains were analyzed for four virulence genes (*toxA*: toxin; *pfhA*: adhesion; *tbpA* and *hgbB*: iron acquisition) by PCR and sequencing and submitted to multilocus sequence typing (MLST).

**Results:**

The eight PmA strains were classified as follows: five as highly pathogenic (HP) for causing necrotic bronchopneumonia and diffuse fibrinous pleuritis and pericarditis; one as low pathogenic for causing only focal bronchopneumonia; and two as nonpathogenic because they did not cause injury to any pig. PCR for the gene *pfh*A was positive for all five HP isolates. Sequencing demonstrated that the *pfh*A region of the HP strains comprised four genes: *tps*B1, *pfh*A1, *tps*B2 and *pfh*A2. The low and nonpathogenic strains did not contain the genes *tps*B2 and *pfh*A2. A deletion of four bases was observed in the *pfh*A gene in the low pathogenic strain, and an insertion of 37 kb of phage DNA was observed in the nonpathogenic strains. MLST clustered the HP isolates in one group and the low and nonpathogenic isolates in another. Only the nonpathogenic isolates matched sequence type 10; the other isolates did not match any type available in the MLST database.

**Conclusions:**

The hypothesis that some PmA strains are primary pathogens and cause disease in pigs without any co-factor was confirmed. The *pfh*A region, comprising the genes *tps*B1, *tps*B2, *pfh*A1 and *pfh*A2, is related to the pathogenicity of PmA. The HP strains can cause necrotic bronchopneumonia, fibrinous pleuritis and pericarditis in pigs and can be identified by PCR amplification of the gene *pfh*A2.

## Background

*Pasteurella multocida* capsular type A (*P. multocida* type A) is one of the most common agents associated with bronchopneumonia in pigs [1]. *P. multocida* type A is usually considered a secondary agent of enzootic pneumonia originally caused by *Mycoplasma hyopneumoniae* (*M. hyopneumoniae*) infection [2, 3]. A few studies have reproduced pneumonia, septicemia or pleurisy in pigs by intranasal or intratracheal challenge, commonly with repeated doses of *P. multocida* [3–5]. According to Ross [6], the difficulty of reproducing the disease in the absence of infectious or noninfectious cofactors is a major limitation to demonstrating the primary role of *P. multocida* type A in pneumonic lesions in pigs. However, our group has successfully developed a model to reproduce the disease in pigs inoculated with a field strain of *P. multocida* type A [7]. This model is useful for studying the pathogenicity of other *P. multocida* type A isolates in the specific pig host.

In this context, Pors et al. [8] previously explored the genetic diversity among isolates of *P. multocida* and its association with pathogenicity. Genetic diversity can be assessed using various DNA-based methods. For *P. multocida* studies, Subaaharan et al. [9] proposed multi-locus sequence typing (MLST). The *toxA*, *tbpA*, *pfhA* and capsule biosynthesis genes have been suggested as epidemiological markers of virulence-associated genes (VAGs) in *P. multocida* field strains [10], and therefore, multiplex PCR for the *toxA*, *tbpA*, *hgbB* and *pfhA* genes was designed for rapid virulence typing [11]. Thus, the objective of the present study was to investigate the capacity of eight *P. multocida* type A field strains to cause disease in healthy pigs.

The *P. multocida* type A strains were screened by PCR for four virulence genes: *toxA,* a protein Gln-deamidating toxin gene; *pfhA,* a filamentous hemagglutinin gene involved in adhesion; and *tbpA* and *hgbB,* transferrin-binding protein and hemoglobin-binding protein genes involved in iron acquisition. These genes were further analyzed by sequencing, and the strains were compared by MLST of seven housekeeping genes (*adk*, *aroA*, *deoD*, *gdhA*, *g6pD*, *mdh*, and *pgi*).

## Methods

### Animals

Seventy-six 120-day-old pigs each weighing 74 kg were used in this study. The animals were derived from a herd with high health status raised at the facilities of the Embrapa Swine and Poultry Research Center. This herd was populated with caesarean-derived colostrum-deprived animals in 2009. Every six months, the pigs are screened for the pathogens described in Table 1, and this procedure was repeated four days before inoculation. Furthermore, respiratory diseases such as enzootic pneumonia, influenza, polyserositis (Glässer disease), atrophic rhinitis, pleuropneumonia and pasteurellosis have never been diagnosed in the herd. The herd is protected by strict biosecurity guidelines and has health barriers that include closed rooms with positive pressure and visitor restriction.

**Table 1:** Semiannual monitoring of pigs from the high-health-status herd: pathogens and laboratory assays

Microorganisms	Samples	Laboratory tests	References
Mycoplasma hyopneumoniae	Tonsillar swabs	Nested PCR	Yamaguti et al. (2008) [43]
	Serum	ELISA	Herdcheck® *M hyo* ELISA – IDEXX
Actinobacillus pleuropneumoniae	Tonsillar and nostril swabs	Bacterial isolation	Quinn et al. (2011) [12]
	Tonsillar swabs	PCR	Souza et al. (2008) [44]
	Serum	ELISA	APP - ApxIV Ab Test - IDEXX
Haemophilus parasuis	Tonsillar and nostril swabs	Bacterial isolation	Quinn et al. (2011) [12]
	Tonsillar swabs	PCR	Redondo et al. (2003) [45]
*Pasteurella multocida*	Tonsillar and nostril swabs	Bacterial isolation	Quinn et al. (2011) [12]
	Tonsillar swabs	PCR	Townsend et al. (2001) [15]
PRRS virus	Serum	ELISA	Herdcheck® X3 PRRS ELISA – IDEXX
Influenza virus^a^	Serum	ELISA	AI Multi-Screen Ab test*®* - IDEXX

^a^Basal levels of circulating antibodies were detected by ELISA. However, genetic material was never detected by RT-PCR

### Animal housing

Four days before inoculation, all animals were transferred from the farm to an isolation unit (biosafety level 2). The animals in each group were housed in different rooms (two pigs per pen) with feed and water provided ad libitum*.* Access to the animals was restricted to the staff. The internal room temperature was monitored daily.

### Strains

Eight strains of *P. multocida* capsular type A from the microorganism collection of the Embrapa Swine and Poultry Research Center were used and are described in Table 2. These strains were isolated from five- to six-month-old pigs with respiratory diseases raised in different herds and were stored in brain-heart infusion broth (BHI; OXOID LTD, Basingstoke, Hampshire, England) with sheep blood (1:1) at − 70 °C until use.

**Table 2:** *Pasteurella multocida* type A isolates used to challenge the pig groups

Strain BRMSA	Group	Brazil State	Brazil Region	Herd production system	Gross lesion
0496	1	Rio Grande do Sul	South	Farrow to finish	Bronchopneumonia
1196	2	Rio Grande do Sul	South	Farrow to finish	Bronchopneumonia
1113	3	Minas Gerais	Southeast	Finisher	Fibrinous pleuritis
1197	4	Rio Grande do Sul	South	Finisher	Bronchopneumonia
1198	5	Santa Catarina	South	Finisher	Bronchopneumonia
1199	6	Paraná	South	Finisher	Necrosuppurative pleuropneumonia
1200	7	Goiás	Midwest	Finisher	Bronchopneumonia
1201	8	Mato Grosso	Midwest	Finisher	Bronchopneumonia

The strains were phenotypically and genotypically confirmed as *P. multocida* type A. The phenotypic characterization was performed according to Quinn et al. [12], and capsular typing was based on acriflavine [13] and hyaluronidase [14] tests. Additionally, all isolates were submitted to species-specific (*kmt*1 gene) multiplex PCR [15] and to capsular typing A (*hya*D-*hya*C) and D (*dcb*F), as described in Table 3.

**Table 3:** Target gene information and primers used for *Pasteurella multocida* type A identification and detection of virulence factors

Gene	Function	Location Gene	N°. Acc.	Primers	DNA-sequences of oligonucleotide primers (5′ – 3′)	Product Size (bp)	Reference
KMT1	Species-specific	213–232	AF016259	KMT1 F	ATCCGCTATTTACCCAGTGG	460	Townsend et al. (2001) [15]
		669–649		KMT1 R	GCTGTAAACGAACTCGCCAC		
hyaD-hyaC	Capsular synthesis	8846–8863	AF067175	CAPA F	TGCCAAAATCGCAGTCAG	1.044	
		9890–9873		CAPA R	TTGCCATCATTGTCAGTG		
dcbF	Capsular synthesis	3142–3165	AF302465.	CAPD F	TTACAAAAGAAAGACTAGGAGCCC	657	
		3789–3766		CAPD R	CATCTACCCACTCAACCATATCAG		
fcbD	Capsular synthesis	2881–2896	AF302467	CAPF F	AATCGGAGAACGCAGAAATCAG	851	
		3733–3714		CAPF R	TTCCGCCGTCAATTACTCTG		
pfhA	Adherence	2409–2427	AY035342	PfhA F	AGCTGATCAAGTGGTGAAC	275	Ewers et al. (2006) [10]
		2684–2665		PfhA R	TGGTACATTGGTGAATGGTG		
tbpA	Iron acquisition	68–85	Pm0337	TbPA F	TTTG GTT GGA AAC GGT AAA GC	728	Ewers et al. (2006) [10] Modified by Atashpaz et al. (2009) [11]
		487–470		TbPA R	TAA CGT GTA CGG AAA AGC CCC		
hgbB	Iron acquisition	308–328	Pm0337	HgbB F	TCA TTG AGT ACG GCT TGA C	499	Atashpaz et al. (2009) [11]
		1096–1077		HgbB R	CTT ACG TCA GTA ACA CTC G		
toxA	Toxin	1878–1897	AF240778	ToxA F	TTCT TAG ATG AGC GAC AAG G	846	Lichtensteiger et al. (1996) [46] modified by Atashpaz et al. (2009) [11]
		2743–2725		ToxA R	GAA TGC CAC ACC TCT ATA G		

### Inoculum

The recovery of *P. multocida* type A from the stock was performed by culture on blood agar plates (Blood Agar Base, BD Difco™, 5% sheep’s blood) incubated at 37 °C for 18–24 h. A subculture on trypticase soy agar (TSA) plates (Difco™) was incubated at 37 °C for 18–24 h. For challenge, bacterial cultures from the third passage were used. The inoculum was prepared with sterile phosphate-buffered saline (PBS) containing 10e7 colony-forming units (CFU)/mL. Serial dilutions and subsequent counting on TSA plates confirmed the inoculum concentration.

### Study design

Eight groups (G1–G8) of eight pigs each were challenged with different strains of *P. multocida* type A. The pigs in the challenged groups (G1-G8) received 3.0 mL (1.5 mL/nostril) of the respective inoculum by slow intranasal dripping in a sitting position. Animals (*n* = 12) in the control group (G0) received 3.0 mL of sterile PBS (1.5 mL/nostril). Each group was housed in a distinct room.

All 76 pigs were clinically evaluated twice a day (0800–0900 h and 1600–1700 h), starting on the 3rd day before the inoculation and continuing until the fifth day post inoculation (5 dpi). The parameters evaluated were rectal body temperature, dyspnea (with animals lying down) and cough (after five minutes of animal movement during feeding and barn cleaning).

### Necropsy

Pigs were euthanized by electrocution, bled and necropsied at 5 dpi. Animals with severe clinical signs were euthanized immediately because of concerns about animal welfare. The lesion features, distribution and severity were recorded at necropsy. The percentage of lung tissue with macroscopic lesions of pneumonia in each lobe was multiplied by each lobe’s relative weight [16]. Pleuritis was classified according to the total affected area using the following scores: 1 (1–25%), 2 (26–50%), 3 (51–75%) and 4 (76–100%). Fragments of the lung, trachea, mediastinal lymph node, heart, pericardial sac, liver, kidney and spleen were preserved in 10% buffered formaldehyde for histopathology and immunohistochemistry. Fragments of the same organs and fibrinous exudates of the pleura, pericardium, peritoneum and joints, whenever present, were collected aseptically and transported to the laboratory at 2–8 °C for bacteriological examination.

### Histopathology and immunohistochemistry (IHC)

Histopathological assays were performed using routine procedures for hematoxylin and eosin staining. Representative slides of each type of lesion were submitted to *P. multocida* type A detection by IHC assay based on the streptavidin-biotin-peroxidase method (LSAB™ System-HRP kit; Dako Cytomation™) and a hyperimmune polyclonal antibody (anti-*P. multocida* type A) produced in sheep. Briefly, tissue fragments with a thickness of 3–5 μm were fixed on poly-L-lysine-treated slides, dewaxed and hydrated. Next, the tissues on the slides were subjected to the following steps: antigen retrieval from tissues by microwave irradiation for 5 min at 700 W, followed by enzymatic digestion with 0.04% pepsin (pH 7.8) for 10 min at 37 °C; blocking of endogenous peroxidase with H_2_O_2_; incubation of the sections with anti-*P. multocida* type A primary sheep polyclonal antibody at a dilution of 1:500 for 2 h at 37 °C; incubation with reagents from an LSAB® HRP Kit (Dako Cytomation®) for 30 min at 37 °C; use of 3-amino-9-ethylcarbazole (AEC) for 5 min at 37 °C; and counterstaining with Mayer’s hematoxylin for 1 min. PBS (pH 7.4) was used for washes between each step.

A lung fragment from a pig previously inoculated with *P. multocida* type A was used as a positive control. A healthy lung fragment was used as a negative control. The results are expressed according to the intensity of the reaction in the lesion, as follows: (−) absence of immunostaining for *P. multocida* type A; (+) mild focal or multifocal areas of staining (up to 25% of the lesion); (++) moderate focal or multifocal areas of staining (26% to 75% of the lesion); and (+++) marked diffuse staining (greater than 75% of the lesion) [17].

To verify the absence of other primary respiratory pathogens, after challenge and necropsy, IHC of porcine circovirus type 2 (PCV2) [18], influenza virus [19] and *M. hyopneumoniae* was performed in all lung samples [7]. The PCV2 test was also performed in mediastinal lymph nodes.

### *Pasteurella multocida* recovery

Samples were plated immediately after collection on blood agar and MacConkey agar (Difco™) and incubated at 37 °C for 24–48 h under aerobic conditions. A streak of *Staphylococcus aureus* was added to an additional plate and incubated microaerophilically at 37 °C for 24–48 h. The biochemical characterization of the isolates was conducted according to Quinn et al. [12].

### Virulence gene profiling

Four virulence-associated genes (VAGs) were assayed by multiplex PCR (Table 3) [11]: *toxA* (a toxin gene), *pfhA* (a gene involved in adhesion), *tbpA* and *hgbB* (genes involved in iron acquisition). DNA was extracted by boiling. The characterization of the target genes and primers and the related references are listed in Table 3. Amplification products were analyzed by gel electrophoresis on a 1.0% agarose gel stained with ethidium bromide and photographed under UV light.

To analyze differences in VAGs and to determine MLST gene relationships, DNA sequencing was performed as follows: 1 ng of DNA was enzymatically fragmented, and libraries were prepared using a Nextera XT DNA Library Prep Kit (Illumina, Inc., San Diego, CA, USA) according to the manufacturer’s recommendations. Library size was evaluated on an Agilent 2100 Bioanalyzer (Agilent Technologies, Santa Clara, CA, USA) and quantified by qPCR. Paired-end sequencing (2 × 250 bp) was performed on an Illumina MiSeq (Illumina, Inc., San Diego, CA, USA) at the Functional Genomics Center, ESALQ, University of São Paulo, Piracicaba, SP. Low-quality reads (phred quality score < 25 and length < 180) and adapters were removed using SeqyClean Software (https://github.com/ibest/seqyclean), reads were assembled using Newbler V. 2.9 (ROCHE), and functional annotation was conducted via the RAST Server (http://rast.nmpdr.org/).

### Multi-locus sequence typing

The genetic relationships of the eight isolates of *P. multocida* were analyzed by sequence alignment of seven housekeeping genes (*adk*, *aroA*, *deoD*, *gdhA*, *g6pD*, *mdh*, and *pgi*) at http://pubmlst.org/pmultocida_multihost. Sequence types and allelic profiles were submitted to the *P. multocida* multi-host MLST database (http://pubmlst.org/pmultocida_multihost/). A neighbor-joining tree was drawn from the concatenated sequences using MEGA 6.0 [20].

### Statistics

The frequency of animals presenting clinical signs, gross lesions and *P. multocida* type A positive isolation in the groups was analyzed with Fisher’s exact test. The group effect on the lung consolidation area (%) was calculated with a Kruskal-Wallis test, followed by a Wilcoxon test for multiple comparisons of groups. The SAS statistical software package version 9.2 [21] was used.

## Results

All animals were negative for respiratory pathogens (*Actinobacillus pleuropneumoniae, Haemophilus parasuis, Bordetella bronchiseptica, M. hyopneumoniae*, PCV2 and influenza virus) when screened before and after inoculation.

### Clinical signs

None of the animals presented clinical signs before challenge. Hyperthermia (rectal temperature ≥ 40 °C) and dyspnea were most frequently observed (Table 4). Higher prevalence and severity of clinical signs were observed in groups G1, G3 and G7 (*p* ≤ 0.001) than in the other groups, starting at six hours after challenge and persisting until euthanasia. The average rectal temperature ± standard error for the animals did not differ (*p >* 0.05) among these challenged groups: 40.44 °C ± 0.11 (G1), 40.42 °C ± 0.19 (G3) and 40.29 °C ± 0.16 (G7), with peaks above 41 °C in some animals. Some pigs from groups G2, G4 and G5 also showed hyperthermia and dyspnea; however, fewer animals were affected, with average rectal temperatures of 39.79 °C ± 0.20, 39.53 °C ± 0.16 and 39.72 °C ± 0.14, respectively. The average rectal temperatures of groups G0, G6 and G8 remained within normal limits (38.97 °C ± 0.06, 39.01 °C ± 0.04 and 39.01 °C ± 0.05, respectively) and did not differ (*p >* 0.05).

**Table 4:** Clinical signs (%) and pathological lesions (%) in pigs challenged with *Pasteurella multocida* type A

Variables	Groups†									p*
	G0	G1	G2	G3	G4	G5	G6	G7	G8	
Clinical signs										
Hyperthermia	0.00^b^	100.0^a^	62.50^ab^	100.0^a^	75.00^ab^	75.00^ab^	12.50^b^	100.0^a^	25.00^b^	< 0.0001
Dyspnea	0.00^c^	100.0^a^	37.50^b^	87.50^ab^	0.00^bc^	25.00^bc^	0.00^bc^	100.0^a^	0.00^bc^	< 0.0001
Cough	16.67^b^	50.00^ab^	37.50^ab^	12.50^b^	0.00^b^	37.50^ab^	0.00^b^	87.50^a^	0.00^b^	0.0002
Macroscopic lesions										
Cranioventral lung consolidation**	0.00^c^	62.50^ab^	37,5,00^ab^	87.50^a^	12.50^b^	0.00^bc^	0.00^bc^	75.00^a^	0.00^bc^	< 0.0001
Cranioventral lung consolidation (%)***	0.00 ± 0.00^b^	3.58 ± 1.51^ab^	2.53 ± 1.43^ab^	6.26 ± 2.38^a^	1.30 ± 1.30^b^	0.00 ± 0.00^b^	0.00 ± 0.00^b^	5.01 ± 1.58^a^	0.000 ± 0.00^b^	0.0004
Necrotic nodules	0.00^c^	62.50^a^	37.50^ab^	75.00^a^	0.00^bc^	0.00^bc^	0.00^bc^	37.50^ab^	0.00^bc^	< 0.0001
Diffuse fibrinous pleuritis	0.00^c^	50.00^ab^	0.00^bc^	75.00^a^	0.00^bc^	25.00^abc^	0.00^bc^	50.00^ab^	0.00^bc^	< 0.0001
Mild focal fibrinous pleuritis	0.00	0.00	0.00	0.00	0.00	12.50	0.00	12.50	0.00	0.7074
Diffuse fibrinous pericarditis	0.00^c^	25.00^abc^	25.00^abc^	62.50^a^	0.00b^c^	25.00^abc^	0.00^bc^	37.50^ab^	0.00^bc^	0.0029
Fibrinous peritonitis	0.00^d^	62.50^a^	12.50^bcd^	37.50^ab^	25.00^abc^	50.00^ab^	0.00^cd^	50.00^ab^	0.00^cd^	< 0.0001
Microscopic lesions										
Fibrinonecrotic suppurative/fibrinonecro-haemorrhagic pleuropneumonia	0.00^c^	87.50^a^	50.00^ab^	87.50^a^	0.00^bc^	0.00^bc^	0.00^bc^	75.00^a^	0.00^bc^	< 0.0001
Fibrinopurulent pleuropneumonia	0.00	0.00	0.00	0.00	12.50	0.00	0.00	12.50	0.00	0.7074
Suppurative lymphadenitis	0.00^c^	12.50^bc^	37.50^ab^	75.00^a^	12.50^bc^	12.50^bc^	0.00b^c^	37.50^ab^	0.00^bc^	0.0005

Fever: Rectal temperature ≥ 40.0 °C

*Descriptive level of probability by Fisher’s exact test; percentages followed by different letters on the same line differ significantly by Fisher’s exact test (*p* ≤ 0.05)

**Suppurative bronchopneumonia in the histopathology assay

***Lung consolidation was measured based on the total percentage of the affected pulmonary area. Descriptive level of Kruskal-Wallis probability; averages followed by different letters differ significantly by the Wilcoxon test (*p* ≤ 0:05)

†G0 with 12 pigs and G1-G8 with eight pigs per group

Coughs were sporadic and of low intensity despite significant differences among the groups (p > 0.05). Furthermore, two pigs (one at 1 dpi and the other at 3 dpi) in G7 had internal otitis, as evidenced by the way the pigs held their heads down. Vomiting was another common clinical sign observed in some pigs of groups G1, G2, G3, G5 and G7 between 1 and 4 dpi. The animals in groups G0 (control), G6 and G8 remained clinically healthy.

### Pathology

**Fig. 1:**
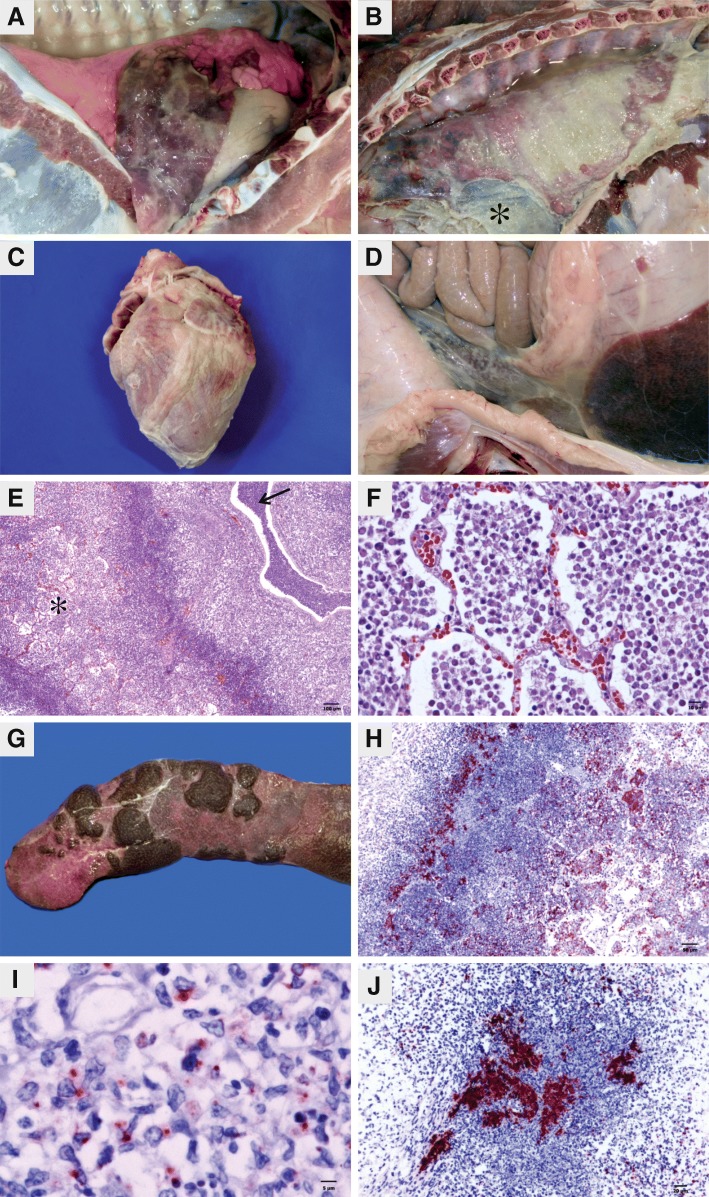
Lesions caused by *Pasteurella multocida* type A in experimentally challenged pigs. **a**. Lung, group 2. Focally extensive hemorrhagic pleuropneumonia in the cardiac lobe with fibrin on the pleura. **b**. Thoracic cavity, group 7. Diffuse fibrinous pleuritis and pericarditis (*). **c**. Heart, group 3. Diffuse fibrinous pericarditis. **d**. Abdominal cavity, group 3. Fibrinous peritonitis. **e**. Lung, group 2. Coagulation necrosis area in the lung parenchyma (*), surrounded by abundant inflammatory cells, mild proliferation of connective tissue and suppurative exudate in the bronchioles **(thin arrow)**. HE. Bar, 100 μm. **f**. Lung, group 2. Abundant (+++) inflammatory exudate, predominantly suppurative, intra-alveolar in a coagulation necrosis area on the lung parenchyma. HE. Bar, 10 μm. **g**. Spleen, group 5. Multiple splenic infarcts with fibrin threads on the capsule. **h**. Lung, group 2. Abundant antigen labeling of *P. multocida* **(red labeling)** in a coagulation necrosis area in the lung and between degenerated inflammatory cells. Bar, 50 μm. **i**. Lung, group 2. Coagulation necrosis area in the lung with *P. multocida* antigen labeling **(red spots)** in the cytoplasm of phagocytic cells. Bar, 5 μm. **j**. Spleen, group 5. Moderate (++) antigen labeling of *P. multocida* **(red labeling)** in a necrotic area. Bar, 20 μm. Immunohistochemistry, streptavidin-biotin-peroxidase method (LSAB™) with 3-amino-9-ethylcarbazole (AEC) and counterstaining with Mayer’s hematoxylin

Because of animal welfare concerns, 19 pigs with severe respiratory clinical signs were euthanized before 5 dpi, as follows: G1 (5/8), G2 (1/8), G3 (6/8), G5 (2/8) and G7 (5/8). All other pigs were euthanized at 5 dpi. The lesions found are described in Table 4. The primary lesions observed according to the frequency and extension were suppurative cranioventral bronchopneumonia, necrosuppurative/necrohemorrhagic fibrinous pleuropneumonia (Fig. 1a), diffuse fibrinous pleuritis (Fig. 1b), fibrinous pericarditis (Fig. 1c), suppurative lymphadenitis and peritonitis (Fig. 1d), which all differed (*p* ≤ 0.05) among the groups.

The average pulmonary consolidation, excluding the necrosuppurative/necrohemorrhagic bronchopneumonial App-like lesion area, was highest in G3 (6.26%), followed by G7 (5.01%), G1 (3.58%), G2 (2.53%) and G4 (1.30%). Histologically, the affected areas had suppurative or fibrinosuppurative bronchopneumonia with abundant neutrophils and bacterial colonies in the lumen of the alveoli, bronchi and bronchioles. Necrosuppurative/necrohemorrhagic fibrinous pleuropneumonia was frequent in pigs from groups G1, G2, G3, G4 and G7, characterized by multifocal areas of coagulation necrosis of the lung parenchyma (Fig. 1e) and associated with suppurative inflammatory exudation (Fig. 1f), proteinaceous material, fibrin, neutrophils, bacterial colonies in the alveolar lumen, necrosis of the vessel walls and possibly hemorrhagic multifocal areas. Fewer oat cells were observed surrounding the necrotic areas in some pigs of groups G3, G4 and G7. The visceral pleura and the adjacent interlobular septa were thickened because of the dilation of lymphatic vessels by fibrin, accumulation of degenerate neutrophils and multiple bacterial colonies. Some lymphatics of the interlobular septa and areas of necrosis were distended and obliterated by fibrin thrombi.

Fibrinous pleuritis was frequently observed but varied in severity among the groups (*p <* 0.0001) (Table 4): 41.94% (13/31) of the occurrences were focal, and 58.06% (18/31) were diffuse. Focal pleuritis occurred unilaterally and was always adjacent to lesions in the lung parenchyma. For diffuse pleuritis, 33.33% (6/18) of the occurrences were unilateral, and 66.66% (12/18) were bilateral. Based on the affected area, pleuritis was scored from 1 to 4, with 1 indicating focal areas of pleuritis and 4 indicating diffuse pleuritis. Diffuse fibrinous pleuritis was observed in pigs from groups G1, G3, G5 and G7, usually associated with fibrinous pericarditis. Of these pigs, one pig each from G1, G2 and G3; three from G5 (with diffuse pleuritis); and another from G5 (with focal pleuritis) had no lung lesions. Histologically, serositis was characterized by thickening of the pleura with accumulation of fibrin, fibroblasts, degenerated neutrophils and multiple bacterial colonies. The pericardial sac was distended by neutrophils and fibrin.

Suppurative lymphadenitis was common except among animals from groups G6 and G8. This lesion was characterized by neutrophils and fibrin and by the distension of the paratrabecular sinus by proteinaceous edema.

Some pigs in groups G1 (*n* = 3), G3 (*n* = 4), G5 (*n* = 1) and G7 (n = 1) had peritonitis. Microthrombi in the spleen were observed in one pig in G1 and two pigs in G5. Furthermore, in one animal in each indicated group, necrotic hepatitis (G1, G3 and G7), splenic necrosis (G1, G5 and G7), splenic infarction (G5: Fig. 1g) and lymphoplasmacytic nephritis (G0, G2, G4, G5 and G8) were observed. Internal otitis was confirmed by the suppurative exudate in both pigs from G7 with clinical signs of otitis.

In summary, *Pasteurella multocida* type A strains were classified by pathogenicity scores according to clinical pathological features primarily produced in inoculated animals, as follows: highly pathogenic strains caused persistent clinical signs and severe lesions consisting of necrosuppurative/necrohemorrhagic bronchopneumonia (App-like) and/or diffuse fibrinous pleuritis and/or pericarditis; low pathogenic strains caused mild clinical signs and only focal bronchopneumonia; nonpathogenic strains did not induce clinical signs, and no lesions were observed. The detailed features of the lesions by strain and pig group are presented in Table 5.

**Table 5:** *Pasteurella multocida* type A strains classification by pathogenicity scores according to clinical pathological features observed in eight challenged pigs per group

Strain BRMSA	Group	Pathogenic classification	Lesions			Euthanasia^b^
			N° of pigs	Area^a^	Predominant features	
0496	1	High	8	3.58	Necrosuppurative/necrohemorrhagic bronchopneumonia (App-like lesion) with diffuse fibrinous pleuritis	5
1196	2	High	4	2.53	Necrosuppurative/necrohemorrhagic bronchopneumonia (App-like lesion) with diffuse fibrinous pleuritis	1
1113	3	High	8	6.26	Necrosuppurative/necrohemorrhagic bronchopneumonia (App-like lesion) with diffuse fibrinous pleuritis and pericarditis	6
1197	4	Low	1	1.30	Focal suppurative bronchopneumonia	0
1198	5	High	3	0.0	Diffuse fibrinous pleuritis, pericarditis and peritonitis	2
1199	6	Nonpathogenic	0	0.0	No lesions	0
1200	7	High	7	5.01	Necrosuppurative/necrohemorrhagic bronchopneumonia (App like lesion) with diffuse fibrinous pleuritis and pericarditis	7
1201	8	Nonpathogenic	0	0.0	No lesions	0

^a^Consolidated lung area excluding App-like lesions, %; ^b^ Number of pigs euthanized before 5 dpi for animal welfare when severe clinical signs were present

### Immunohistochemistry

Marked diffuse staining (+++) of *P. multocida* was detected by IHC in the fibrinosuppurative exudate of the pleura and pericardium, in areas of necrosuppurative bronchopneumonia (Fig. 1h), and in the the cytoplasm of the majority of lymphocytic cells (Fig. 1i) but also free in inflammatory exudates. Mild (+) to moderate (++) multifocal staining was observed in the exudate in the lumen of the bronchi and bronchioles and in the interlobular septa in areas of suppurative bronchopneumonia. Moderate (++) *P. multocida* staining was observed in the cytoplasm of the macrophages in the necrotic exudate within the crypts of the tonsils and in macrophages and neutrophils of the mediastinal lymph nodes. *P. multocida* antigen labeling was mild and multifocal (+) in the kidney, liver and spleen macrophages. Moderate and multifocal (++) *P. multocida* antigen labeling was observed in a septic infarction area in the spleen (Fig. 1j). Additionally, mild (+) staining of *P. multocida* was visualized in the lumen of vessels in the lung, heart, mediastinal lymph nodes and spleen of some pigs.

### Recovery of *Pasteurella multocida*

The isolation of *P. multocida* type A from tissues differed among groups (*p* ≤ 0.05, Table 6), with the highest frequency in pigs from G1 (100%), G3 (100%) and G7 (87.5%). The recovery of *P. multocida* type A from the lung, pericardial sac, pleura, trachea, mediastinal lymph nodes and peritoneum differed among groups (p ≤ 0.05), with the highest recovery in the thoracic cavity from tissue lesions in pigs from G1, G2, G3, G4, G5 and G7. *Pasteurella multocida* type A was recovered outside the thoracic cavity in 13 pigs. The sites were the peritoneum cavity, liver, spleen and kidney in animals from G1, G3, G5 and G7; the femoral-tibial-tarsal joint in two animals (G5 and G7); and the purulent exudate of the inner ear of two pigs from G7 with clinical signs of otitis. Bacterial growth was not obtained from animals in groups G0, G6 and G8.

**Table 6:** Frequency of *P. multocida* type A recovery from different organs per group of pigs

Samples	Groups									*p* ^a^
	G0	G1	G2	G3	G4	G5	G6	G7	G8	
N° of animals challenged	12	8	8	8	8	8	8	8	8	
Whole Animal	0^d^	8^a^	3^bc^	8^a^	1^cd^	3^bc^	0^cd^	7^ab^	0^cd^	< 0.0001
Lung	0^c^	7^a^	3^ab^	7^a^	1^bc^	3^ab^	0^bc^	7^a^	0^bc^	< 0.0001
Pericardium	0^b^	1^ab^	0^ab^	4^a^	0^ab^	2^ab^	0^ab^	4^a^	0^ab^	0.0009
Pleura	0^d^	5^ab^	1^bcd^	7^a^	0^cd^	2^bcd^	0^cd^	4^abc^	0^cd^	< 0.0001
Trachea	0^b^	3^a^	1^ab^	4^a^	1^ab^	2^ab^	0^ab^	2^ab^	0^ab^	0.0383
Mediastinal lymph node	0^bc^	5^ab^	2^b^	8^a^	0^b^	2^b^	0^b^	3^b^	0^b^	< 0.0001
Peritoneum	0^b^	3^a^	0^ab^	4^a^	0^ab^	1^ab^	0^ab^	1^ab^	0^ab^	0.0039
Spleen	0	2	0	1	0	1	0	1	0	0.3717
Liver	0	2	0	1	0	1	0	1	0	0.3717
Kidney	0	2	0	1	0	2	0	0	0	0.1188
Joint	0	0	0	0	0	1	0	1	0	0.7074

letters on the same line differ significantly by Fisher’s exact test (*p* ≤ 0.05)

^a^Descriptive level of probability by Fisher’s exact test; percentages followed by different

### Genotypic characterization

The identity of *P. multocida* was confirmed by PCR detection of the *kmt* gene (species- and type-specific) in all tested isolates. Capsular type A was confirmed by detection of the *hyaD-hyaC* gene in association with a hyaluronidase-positive test and negative acriflavine precipitation. Moreover, the *hgbB* gene was detected in all eight isolates used in the challenge, and the *tbpA* and *toxA* genes were not detected. The *pfhA* gene was detected by PCR exclusively in all the high-pathogenicity isolates.

**Fig. 2:**
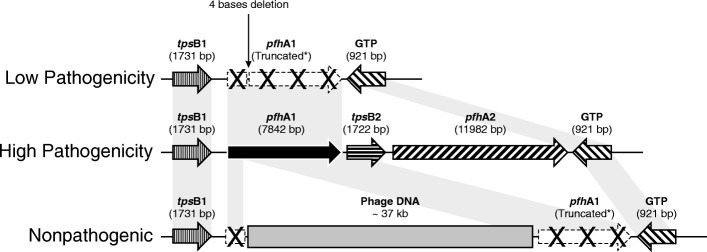
Schematic representation of the *pfh*A gene region of *P. multocida* type A according to pathogenic classification

Consequently, the sequence analysis focused on the region of the *pfhA* gene of all eight isolates. The *pfhA* region of all five highly pathogenic strains consisted of four genes: tpsB1 (1731 bp); *pfhA1* (7842 bp); tpsB2 (1722 bp); and *pfhA2* (11,982 bp). The single low pathogenic strain did not have the tpsB2 and *pfhA2* genes, in addition to presenting a four-base deletion in the beginning of the *pfhA1* gene (at position 892–895 bp) that caused a frameshift and a premature stop codon. The two nonpathogenic isolates also did not have the tpsB2 and *pfhA2* genes and contained a phage DNA insertion of 37 kb located within the *pfhA1* gene (Fig. 2).

**Fig. 3:**
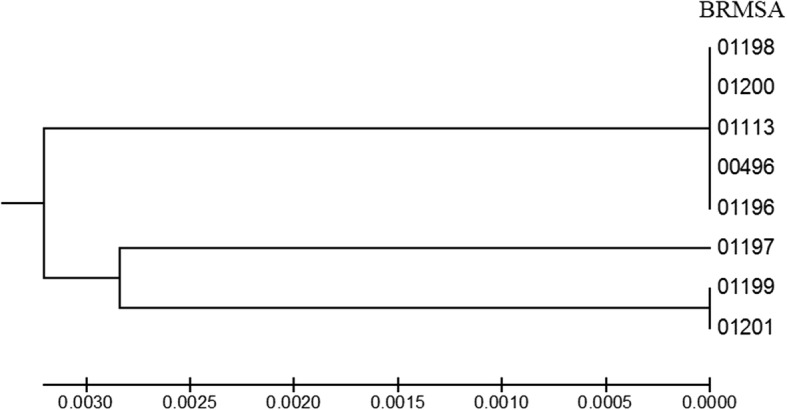
Dendrogram representative of the 7 concatenated gene sequences used for the MLST of the 8 isolates of *P. multocida* type A. The MLST was generated by joint analysis of seven housekeeping genes (*adk*, *aroA*, *deoD*, *gdhA*, *g6pD*, *mdh*, and *pgi*) using the RIRDC MLST database

MLST profiles and phylogenetic analysis classified the eight isolates into two groups, G1 and G2 (Fig. 3). **G1** contained the five highly pathogenic isolates. **G2** was composed of the low pathogenic strain (G 2.1) and the nonpathogenic strain (G 2.2). The G1 and G.2.1 isolates did not present a corresponding sequence type (ST) in the MLST database for the seven housekeeping genes analyzed. The isolates of the G.2.2 group showed 100% similarity to ST 10.

## Discussion

This study successfully demonstrated that some strains of *P. multocida* type A could induce bronchopneumonia, serositis and septicemia in pigs without interference from other pathogens. Furthermore, two strains were categorized as nonpathogenic. Outbreaks of severe respiratory disease in finishing pigs, characterized by fever, dyspnea, serositis and bronchopneumonia, frequently occur in Brazilian pig production herds. *Pasteurella multocida* type A has been isolated from these outbreaks, associated or not associated with other agents. In some cases, hemorrhagic necrotic foci of pleuropneumonia were found by practitioners in Brazil and named “*A. pleuropneumoniae-*like” because of their similarity to lesions caused by infection with *A. pleuropneumoniae*. Traditionally, *P. multocida* is regarded as a secondary opportunist in the Porcine Respiratory Disease Complex (PRDC) [1–3, 22], and descriptions of cases in which *P. multocida* is suspected to act as a primary agent of lung infection are scarce [4].

Considering the clinical and pathological results obtained with the challenged animals, the isolates were classified into three pathogenic categories: highly pathogenic, low pathogenic and nonpathogenic. The primary observed clinical signs were hyperthermia and dyspnea. Isolates considered nonpathogenic did not primarily induce the disease in healthy pigs; however, these isolates may have an important role in the PRDC [2, 23].

Suppurative/fibrinosuppurative bronchopneumonia, necrosuppurative/necrohemorrhagic pleuropneumonia, pleuritis and pericarditis were the primary observed lesions, similar to the results of other studies of pigs challenged with *P. multocida* type A [4, 24]. In the present study, lung consolidation was not extensive, but *P. multocida* type A was recovered and visualized in low to moderate amounts in the exudates of the bronchi, bronchiolar lumens and interlobular septa. Therefore, *P. multocida* type A is likely not the primary agent in extensive consolidations of lung lesions with mixed infection, as observed in the PRDC [23]. The clinical pathological picture observed in some challenged groups was similar to that of a cattle disease caused by *Mannheimia haemolytica* (family *Pasteurellaceae*) secondary infection triggered by predisposing factors [25].

Some challenged pigs presented peritonitis concurrently with pleuritis and pericarditis, demonstrating bacterial tropism for the serous membranes. *Pasteurella multocida* type A is frequently associated with pleuritis in slaughtered pigs [26]. In this study, severe and diffuse pleuritis and pericarditis were observed in many challenged groups. These findings are important because they suggest that *P. multocida* type A is one of the causes of chronic pleuritis and pericarditis observed at slaughter. Additionally, some pigs only presented fibrinous pleuritis, whether associated with fibrinous pericarditis or not, without lung parenchyma injuries. Such lesions are similar to those caused by *H. parasuis* in Glässer disease [27]. Therefore, in clinical cases of bronchopneumonia and serositis, confirmation of the etiological diagnosis with laboratory tests is essential, particularly in the presence of pulmonary necrotic nodules, pleuritis and pericarditis.

*P. multocida* type A has been commonly isolated as a secondary pathogen from respiratory lesions caused primarily by *M. hyopneumoniae* or influenza A [1–3]. In 2004, Cappuccio et al. [24] described a new form of severe infection by *P. multocida* type A that caused severe fibrinous and necrohemorrhagic lung lesions in pigs on farms in Argentina. Subsequently, a similar disease was observed in Brazil by Embrapa researchers. Necrosuppurative/necrohemorrhagic pleuropneumonia occurred in some challenged pigs with intense *P. multocida* labeling by IHC in these areas. These lesions were called “*A. pleuropneumoniae*-like” or “App-like” lesions due to their similarity to those produced by *A. pleuropneumoniae* [28]. Notably, as described above, the lesions caused by *P. multocida* type A are similar to those produced by other agents of the family *Pasteurellaceae*, such as *H. parasuis*, *A. pleuropneumoniae* and *M. haemolytica.*

Septicemic forms of *P. multocida* infections have been reported to be characteristic of capsular serotype B, affecting birds, buffalo and pigs [29]. However, septicemic forms with capsular serotype A have also been described in pigs [4, 5, 30, 31]. In this study, 13 pigs had a septicemic form. *Pasteurella multocida* type A was isolated and visualized in several parenchymal organs and in serositis (Table 4). The VAGs associated with the pathogenesis of pneumonia and pleuritis caused by *P. multocida* infection are not clear [32]. The lipopolysaccharide of the bacterial cell wall may have an important role in the induction of the inflammatory response of the host [33]. Additionally, the *P. multocida* capsule is clearly involved in the evasion of phagocytosis and complement resistance [34]. However, information related to the invasiveness of *P. multocida* is limited. The mechanisms of mucosal resistance to innate immunity and how these mechanisms cause systemic lesions have not been clearly elucidated.

The genetic diversity among *P. multocida* isolates obtained from cases of pneumonia in pigs has been investigated using different molecular techniques [30, 35, 36]. This study demonstrated differences in *pfhA* PCR results; only highly pathogenic strains were positive. Additionally, the sequence analysis revealed that the PCR target region (*pfh*A2*)* was absent in the low pathogenic and nonpathogenic isolates. These results support the importance of the multiplex PCR technique [11] for virulence genes and the *pfh*A2 gene as a possible marker of high pathogenicity.

The sequence of the *pfh*A region of highly pathogenic strains is composed of four genes (*tps*B1, *Pfh*A1, *tps*B2 and *Pfh*A2). A similar system including two potential virulence CDs and their accessory genes was previously described by May et al. [37] for the Pm70 strain isolated from fowl cholera. By contrast, as shown in Fig. 2, the low and nonpathogenic strains did not have the genes *tps*B2 and *Pfh*A2. Additionally, a four-base deletion and a 37 kb phage DNA insertion in *pfh*A1 were observed in the low and nonpathogenic strains, respectively.

The *pfh*A gene encodes filamentous hemagglutinin, an adhesin required for bacterial colonization in the upper respiratory system [38]. The PCR results and gene sequencing information demonstrated the absence of *pfh*A2 and a four-base deletion or phage insertion within *pfh*A1. These findings could help explain the reduction or elimination of pathogenic features. Specifically, for the low pathogenic strain, the inactivation of the *pfh*A1 gene by a premature stop codon did not eliminate pathogenicity; thus, other genes are most likely involved in the pathogenic process. Gene inactivation of *P. multocida pfh*A was previously related to colonization ability in the upper respiratory tract in turkey [39] and mouse [40]. The associated *tps*B gene belongs to the TpsB family, which is responsible for transporting sugars (trehalose) to the cell and is therefore related to cellular structure [41].

MLST was previously used to demonstrate clonal differences among *P. multocida* isolates [6, 42]; however, Pors et al. [8] did not find a relationship between MLST results and pulmonary lesions. In this study, MLST differentiated the isolates according to the pathogenic features demonstrated in the experiment. The five highly pathogenic isolates in this analysis originating from 4 different Brazilian states were identical, demonstrating that the highly pathogenic strains are widespread in pig production areas in Brazil. The low pathogenic and nonpathogenic isolates were clustered together in another group with small differences between them. In the MLST analysis, the nonpathogenic isolates were also identical. In addition, only the nonpathogenic isolates were assigned a sequence type (ST10) when submitted to MLST analysis. Of the 17 strains belonging to ST10 deposited in the MLST database, 11 were isolated from pigs, and 8 of these were from pneumonia. By contrast, the high and low pathogenicity isolates were not similar to any strains for the seven housekeeping genes analyzed. These results reinforce our hypothesis that the genetic variability among *P. multocida* type A strains is related to pathogenicity in pigs.

## Conclusions

The hypothesis that some *P. multocida* type A strains are primary pathogens and cause disease in pigs without any co-factor was confirmed. The *pfh*A region, consisting of the genes *tps*B1, *tps*B2, *pfh*A1 and *pfh*A2, is related to the pathogenicity of *P. multocida* type A. The highly pathogenic strains produce necrotic bronchopneumonia, fibrinous pleuritis and pericarditis in pigs and can be identified by PCR of the *pfh*A2 gene.

## Abbreviations

A. pleuropneumoniae: Actinobacillus pleuropneumoniae
AEC: 3-amino-9-ethylcarbazole
B. bronchiseptica: Bordetella bronchiseptica
BHI: Brain-heart infusion broth
BRMSA: Brasil Microrganismo Suínos e Aves
CFU: Colony-forming units
dpi: day (s) post inoculation
G1-G8: Experimental groups
H. parasuis: Haemophilus parasuis
HP: Highly pathogenic
*M. hyopneumoniae*: Mycoplasma hyopneumoniae
MLST: Multilocus sequence typing
PBS: Phosphate-buffered saline
PCR: Polymerase chain reaction
PCV2: Porcine circovirus type 2
PmA: *Pasteurella multocida* Type A
PRDC: Porcine respiratory disease complex
qPCR: Quantitative PCR
RAST: Rapid Annotation using Subsystem Technology
TSA: Trypticase soy agar
VAGs: Virulence-associated genes
